# Supplementary material for: Splice-Junction-Based Mapping of Alternative Isoforms in the Human Proteome
Source: Cell Rep. Author manuscript; Available in PMC 2020 Jan 15. (PMC6961840; doi:10.1016/j.celrep.2019.11.026)

A

sp|Q8WZ42|TITIN_HUMAN|ENSG00000155657|SE2|14023|chr2|178622767|178624731|−0|r960|T1
THANLFVKAPHVEFLRPL q value: 0.001774 Tr_novel:TRUE RefSeq_Novel:TRUE
Search result spec prec mz: 523.5506 Actual spec prec mz: 523.5506
Fragments matched per AA: 1.06 Proportion of top 20 peaks matched: 0.4

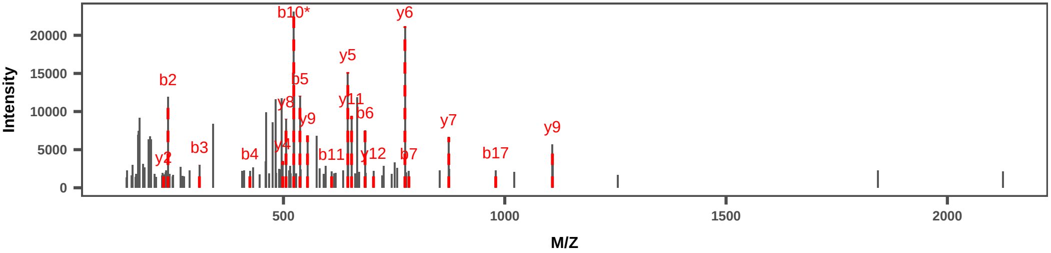

B

Scatterplot of predicted elution time
Fitting R2: 0.774
Novel peptide residual Z score: −2.03
Number of peptides: 1747

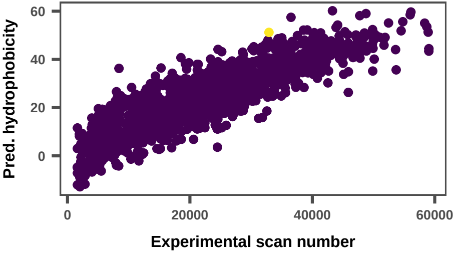

C

Distributions of residuals from best−fit line
of predicted RT vs Expt. scan number
Line: Z score of novel peptide
Z: −2.03

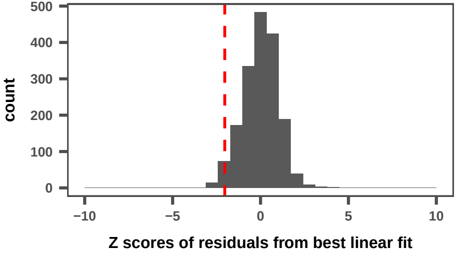

Supplement: 2 [file NIHMS1546469-supplement-2.zip › DF1/PXD010154/Heart/Heart_70_TTN_THANLFVKAPHVEFLRPL.pdf]
